# Lessons to be learned from an attempted RCT: iCHIMPS—an online intervention for adolescents with mentally Ill parents

**DOI:** 10.3389/fdgth.2025.1526995

**Published:** 2025-07-25

**Authors:** P. Dülsen, K. Barck, S. Wiegand-Grefe, A. Leidger, T. Paumen, H. Baumeister

**Affiliations:** ^1^ Department of Clinical Psychology and Psychotherapy, Institute of Psychology and Education, Ulm University, Ulm, Germany; ^2^ Department of Psychiatry and Psychotherapy, University Medical Centre Hamburg-Eppendorf, Hamburg, Germany

**Keywords:** Internet- and mobile-based intervention, e-health, online intervention, children and adolescents, mentally ill parents

## Abstract

**Background:**

Children and adolescents with mentally ill parents represent an at-risk population for developing mental disorders themselves. Internet- and mobile-based interventions (IMIs) have been demonstrated to be an effective, scalable, and temporally and geographically independent method of treatment delivery. However, evidence for IMIs aimed at children and adolescents remains limited and inconclusive, especially for children of mentally ill parents. Therefore, the present trial aimed to evaluate the effectiveness of a mental health IMI (iCHIMPS) for children of parents with a mental illness. Due to insufficient recruitment, however, this article will primarily focus on lessons learned from the challenges encountered during the study’s implementation.

**Methods:**

The IMI was targeted at children aged 12–18 years, regardless of whether they exhibited symptoms of mental disorders, provided that at least one parent had a diagnosed mental illness. To evaluate the effectiveness, the IMI was provided to one group [intervention group (IG)] while the control group received treatment as usual (TAU). At four measurement timepoints, the primary outcome (Youth Self-Report—YSR 11-18R) and various secondary outcomes were assessed. Recruitment from May 2021 to April 2023 initially took place at 21 participating mental health clinics throughout Germany and was later supplemented by various additional clinics as well as recruitment pathways.

**Results:**

In total, *n* = 22 participants were recruited. This result was far off the needed number of participants to meaningfully conduct any analyses. Therefore, no quantitative analyses were conducted, and this trial is discussed as a failed trial, providing important insights into ineffective strategies for reaching adolescents of parents with mental illnesses, in particular, and adolescents through digital interventions more generally.

**Conclusion:**

The identified reasons for the failed recruitment include the complex study design, particularly the presence of multiple concurrent trials recruiting from the same population, the inherent difficulty of reaching families with mentally ill parents, and the limitation of targeting the IMI solely at adolescents rather than involving families more broadly. Additionally, the design may not have been sufficiently engaging or appealing to adolescents. These reasons are discussed along with the implications for future IMI research involving children and adolescents.

**Clinical Trial Registration:**

identifier (DRKS00025158).

## 1 Introduction

Internet- and mobile-based interventions (IMIs) have previously been demonstrated to be effective, scalable, and flexible in their use for various mental disorders in adults and youths (1–7). However, the current evidence for children and adolescents remains inconclusive. IMIs for children have been studied only infrequently, while IMIs for adolescents have shown mixed results, with some studies reporting non-significant pooled effects when compared with both passive and active control groups (2, 3, 5, 8).

One population that has clearly received insufficient attention and has been identified as at-risk comprises children and adolescents of mentally ill parents. An estimated 25% of children or adolescents belong to this group due to having at least one parent with at least one mental disorder (9–13). Research indicates that >50% of these children may develop any mental disorder themselves (14, 15). The risk increases notably with age and varies depending on both the type of disorder in the parent and the specific disorder in the child (15). Depending on these factors, children of parents with mental illness may have a 2–8 times higher likelihood of developing symptoms of mental disorders compared with that of children whose parents do not have a mental illness (10, 14–16). IMIs targeting children and adolescents with mentally ill parents are very limited and show mixed findings (17–21). Two pilot trials with participants aged 18–25 years reported a reduction in depression and stress symptoms (19) or anxiety, as well as improved coping and self-efficacy (17). However, another trial for individuals aged 16–25 years reported no effect of their intervention (21).

As a subproject of the joint multicenter project CHIldren of Mentally ill ParentS-NETwork (CHIMPS-NET), the present RCT evaluated an IMI named iCHIMPS for its clinical and cost-effectiveness in promoting mental health. iCHIMPS was designed for adolescents aged 12–18 years with parents having mental disorders, regardless of whether the adolescents already exhibited signs of mental disorders themselves or not. The RCT aimed to explore the following:

1. The clinical effectiveness of iCHIMPS compared with TAU (treatment as usual) regarding mental health at 6-month follow-up
2. The clinical effectiveness of iCHIMPS compared with TAU (treatment as usual) regarding the secondary outcomes (mental wellbeing, further mental health outcomes, self-efficacy, and coping strategies)
3. Moderators and mediators of intervention success, as well as potential adverse events

The randomized controlled trial (RCT) was launched with clearly defined aims, but recruitment ultimately fell short of expectations—a point that will be addressed in detail in the following sections. While the trial was completed, it failed to meet its recruitment targets, and the results presented represent only a fraction of what was originally planned. Accordingly, the primary focus of this article shifts to a critical examination of the barriers that impede successful recruitment. This includes placing our experience within the broader context of existing research and discussing the implications for future intervention studies targeting children and adolescents affected by parental mental illness. By reflecting on the challenges encountered, this article aims to offer meaningful insights to guide the design and implementation of future research in this complex and sensitive area.

## 2 Materials and method

### 2.1 Study design

The present study is based on a two-armed, multicenter, cluster-randomized controlled trial (cRCT) comparing the clinical effectiveness and cost-effectiveness of using the iCHIMPS IMI between the intervention group (IG) and a treatment-as-usual (TAU) control group.

The iCHIMPS study was reviewed and approved by the local ethics committee of Ulm University, Germany (189/20-FSt/bal.), and was registered in the German clinical trial register (DRKS00025158). All results will be reported in accordance with the Consolidated Standards of Reporting Trials (CONSORT) Statement 2010 as well as with the extensions for reporting cluster-randomized trials and trials on psychological interventions (22, 23).

A study protocol (24) outlining the whole study in detail according to SPIRIT guidelines (25) has been previously published and is used as a template for the present more concise version of the method section.

### 2.2 Intervention

The intervention was developed by integrating existing Internet- and mobile-based programs (26) with content from the CHIMPS program (27). All material was adapted to be age-appropriate, as only children and adolescents would use the intervention. To enhance accessibility, key content was presented through videos and animations. Adolescents then tested a beta version in think-aloud sessions and provided feedback, which primarily concerned the length of the modules and the text-heavy nature of the content. These insights were used to refine the intervention, which was subsequently finalized and uploaded to the digital platform. However, the intervention remained predominantly text-based, with some sections that might still be perceived as overly lengthy. This problem will be addressed later in the article. The intervention was created for adolescents who either already showed symptoms of mental disorders or did not, and was designed in a way to be used by them alone without parental supervision. The main goals of the iCHIMPS intervention are to improve mental health, quality of life, and self-management abilities as well as to enable the adolescents to better deal with their complex life situations. iCHIMPS consisted of eight consecutive modules, including text, picture, video, and audio formats, and the design followed persuasive principles (28, 29), with motivational messages, reminders, and appointments for when the next module would be started. Covered topics include engagement with the users’ own life situation, their own challenges, resources, and strengths, psychoeducational content of the mental disorder of their parent(s), health, communicating about difficult topics, strengthening familial and social relationships, stress management, emotion regulation, and establishing healthy boundaries to increase autonomy. iCHIMPS was accessible through the Internet from any computer, laptop, or smartphone. The theoretical background of the intervention was based on a psychodynamic therapy approach (26, 27) and a cognitive behavioral approach. To facilitate the transfer of what the participants learned during the intervention into their daily lives, the intervention provided interactive home tasks and helpful suggestions in terms of take-home messages. Additionally, e-coaches were available to support and guide the participants through individualized feedback and answer questions asynchronously.

The iCHIMPS intervention was hosted through eSano, an open-source e-health platform developed by the Department of Clinical Psychology and Psychotherapy and the Institute of Databases and Information Systems at Ulm University (30, 31). The platform was developed considering the IEC 62304 (safety class B), the GAMP5 (category 4), the General Principles of Software Validation of the FDA, and the Pharmaceutical Inspection Cooperation Scheme (PIC/S) 11-3.

### 2.3 Procedure

If a potential family recruited by the study staff at the participating clinics met all previously mentioned inclusion criteria, as well as agreed to participate in the iCHIMPS intervention and not in one of the three other intervention arms, a clinical assessment interview was conducted. After the interview, the recruitment at the clinics was finalized, and the online baseline assessment (t0) had to be filled out by all participating family members. If all criteria were still fulfilled, the whole family, as a cluster, was randomized based on block-wise randomization with a variable block length and without stratification. If the participant was assigned to the iCHIMPS intervention group, he or she was able to complete the eight consecutive modules of the Internet-based intervention available at https://patient.esano-trainings.de. All participating family members of both groups received emails with invitations to the online assessments for t1 (1-month post-inclusion), t2 (2-month post-inclusion), and t3 (6-month post-inclusion). The participating adolescents were compensated for their participation after the successful completion of the online assessment at t2 and t3 with 20 € for each assessment.

Due to recruitment problems in the whole CHIMPS-NET project, to a large extend, but not solely, caused by the COVID-19 pandemic, the 21 adult mental health clinics, as the sole recruitment locations, were successively complemented by additional clinics, psychiatrists, psychotherapists, self-help groups, schools, online advertisements, and many more additional recruiting strategies and locations. The inclusion and exclusion criteria remained unchanged throughout.

### 2.4 Participants

The clusters under study were families, which were defined as (a) having at least one parent with a mental disorder according to the ICD-10 within the last 6 months and (b) at least one child between 12 and 18 years of age. Further inclusion criteria were as follows: all participating family members needed to (c) have access to the Internet on a computer, laptop, or smartphone, be able to sufficiently understand the German language, and had to (d) have agreed to the informed consent. Exclusion criteria included (a) acute suicidal tendencies, (b) acute substance use disorder (ICD F1X.2 except nicotine dependence F17.2), or (c) acute psychotic symptoms by the adolescent(s).

Hereafter, the participating adolescents are referred to as participants, while participating parent(s) will be called parents.

### 2.5 Recruitment

Participant recruitment was conducted over a 2-year period, from 1 May 2021 to 30 April 2023. Initially, recruitment efforts were concentrated within 21 participating adult mental health clinics across Germany. At each site, trained study personnel systematically engaged with clinic staff across different departments to identify adult patients who were parents of minor children—our primary target group.

The recruitment process was designed to follow a parent-to-child pathway. Once eligible parents were identified by clinic staff, they were approached by the study team and provided with preliminary information about the study. If interest was expressed, families were invited to attend an initial informational session, during which detailed study procedures, inclusion criteria, and ethical considerations were discussed. During this session, the entire study, including all of its separate intervention arms, was introduced: three in-person interventions [differentiated by the severity of presented symptoms (32)] and one online intervention. Written informed consent was obtained from all participating adults and, where appropriate, assent from children.

Despite these efforts, it became apparent that the initial recruitment strategy alone would not suffice to achieve the projected sample size. As a result, the recruitment protocol was expanded to incorporate a broader set of strategies and access points. Additional mental health clinics were contacted, and outreach efforts extended to various offline and online settings. These included distributing printed materials (e.g., flyers and posters), publishing study announcements in clinic newsletters, engaging with self-help and family support groups, and launching targeted social media campaigns.

Given the limited response through the original parent-to-child pathway, we eventually adapted the protocol to include child-to-parent recruitment channels. Although this was not planned initially, it became necessary to reach the desired sample size. In this phase, outreach was conducted directly through schools, youth social services, child welfare agencies, and school social workers. Children and their caregivers were informed about the study through in-person presentations, school newsletters, and expanded social media outreach tailored to youth and family audiences.

Through this multitiered, adaptive recruitment approach, we aimed to ensure broad visibility of the study and maximize the diversity and representativeness of the sample.

### 2.6 Outcomes

The primary outcome measure was the mental health of the participant at 6-month post-inclusion (t3) as measured by the Youth Self-Report score [YSR 11-18R; (33)]. All other outcome measures and further measurement details are provided within the study protocol (24).

A special focus within this article is also given to the assessment of potential adverse effects. The Negative Effects Questionnaire [NEQ; (34)] was used to evaluate negative outcomes that may have arisen during the intervention. This self-report tool captures negative experiences participants may attribute to the intervention, such as increased stress, emotional discomfort, or interpersonal difficulties. It also differentiates between effects caused by the intervention itself and those resulting from external factors, thereby supporting ethical monitoring and ensuring participant safety.

### 2.7 Data management

Since no statistical analyses were performed, we do not report the planned analyses here. However, all analyses were defined *ad hoc* and can be found in the study protocol (24).

Negative effects were evaluated descriptively by summarizing the frequency and proportion of reported negative effects, along with participants' attribution to the intervention. Additionally, items were thematically combined to try to identify recurring concerns and contextual factors. However, the combination of items follows a strict exploratory approach and is not founded in the existing literature of the scale. This combined analysis provided a nuanced understanding of potential intervention-related risks.

Dropouts are categorized as follows: study dropouts leave the study entirely, assessment dropouts stop completing evaluations but may stay in treatment, and intervention dropouts discontinue the treatment but may still complete assessments. This distinction clarifies where disengagement occurs.

## 3 Results

### 3.1 Recruitment

**Figure 1:**
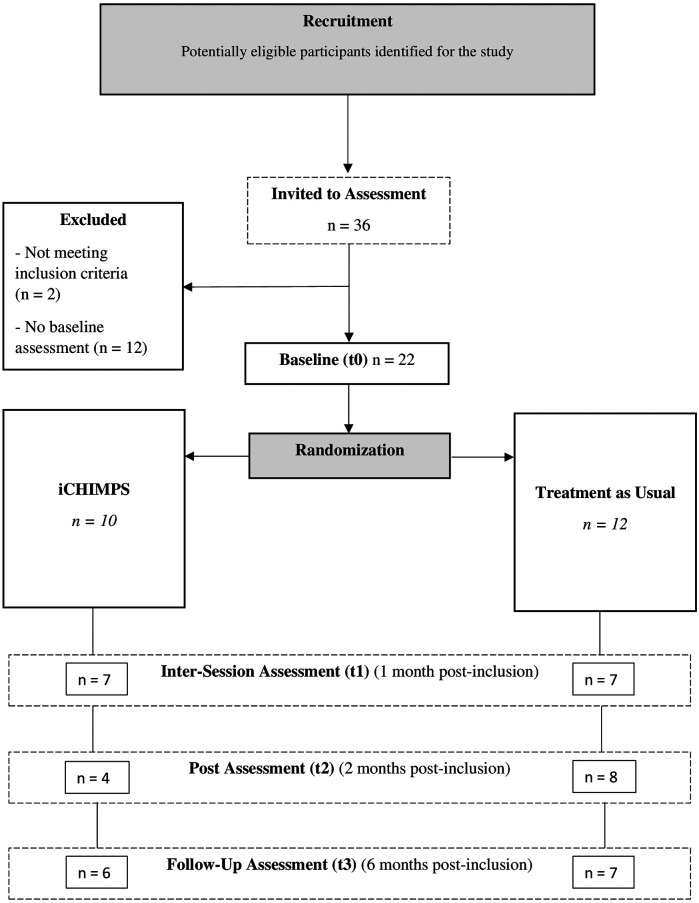
Flowchart.

In total, *n* = 22 participants from 20 family clusters were randomized into the iCHIMPS intervention group (*n* = 10) and the TAU control group (*n* = 12). Thirty-six individual participants were invited to the online assessment, *n* = 2 were excluded due to not meeting the inclusion criteria, and *n* = 12 did not complete their baseline online assessment. Intersession assessment (t1) was completed by *n* = 14 (63.64%), post-assessment (t2) was completed by *n* = 12 (54.55%), and follow-up assessment (t3) was completed by *n* = 13 (59.09%). Participant flow is illustrated in Figure 1. The trial had to be terminated prematurely after 2 years due to insufficient recruitment success.

The recruited families for iCHIMPS were primarily reached through adult mental health clinics (*n* = 20, 90.9%) and, to a lesser extent, via social media (*n* = 2, 9.1%), based on a total of 22 participating families. Recruitment for iCHIMPS began approximately 6 months after the start of recruitment for the other interventions within the CHIMPS-NET project, and iCHIMPS was often presented only after families declined participation in one of the other intervention options. The number of families contacted during recruitment, for all interventions or any individual intervention, cannot be accurately determined, as no standardized tracking procedure was implemented. Nonetheless, recruitment efforts were extensive, involving numerous staff hours and multiple pathways over a 2-year period. While this limitation prevents valid quantification, it should not diminish the substantial operational effort invested throughout the recruitment phase.

Specifically, for iCHIMPS, however, some adolescents had voiced to the study staff during the recruitment that they are not the ones having the problems and, therefore, did not understand why they should participate in an intervention.

On average, the participants were 17.02 years old (SD = 2.05) and ranged from 14.2 to 18.18 years. Gender was almost equally distributed, female participants *n* = 12 (54.55%) and male participants *n* = 10 (45.45%). A majority of participants were living with divorced parents *n* = 13 (59.15%), most participants were still in school *n* = 12 (54.55%), and a majority of participants had specified to qualify for a mental disorder *n* = 12 (54.55%). For *n* = 14 participants, only the mother (63.63%); for *n* = 4 participants, only the father (18.18%); and for *n* = 4 participants, both parents had at least one mental disorder (18.18%). The most common disorder among all participants was depression, affecting five individuals (41.67%). Among mothers, depression was also the most prevalent, reported by nine (50%), followed by PTSD, which affected five (27.78%). Among fathers, three (37.5%) were diagnosed with depression. Descriptive statistics can be found in Tables 1–4.

**Table 1:** Sociodemographic characteristics.

Variable	*n*	%
Gender		
Female	12	54.55
Male	10	45.45
Marital status of parents		
Single	1	4.55
In relationship, living apart	1	4.55
Married, living together	6	27.3
Divorced, living apart	13	59.15
Widowed	1	4.55
Siblings		
None	4	18.2
One	8	36.4
Two	7	31.85
Three	3	13.65
School degree		
No school degree	4	18.2
Still in school	12	54.55
Lower secondary school degree	1	4.55
Secondary school degree	3	13.65
Advanced technical college entrance qualification	1	4.55
Other	1	4.55
Vocational qualification		
None	18	81.9
Still in vocational training/college education	4	18.2
Mental disorders		
Youth	12	54.55
Mother	14	63.63
Father	4	18.18
Mother and father	4	18.18

**Table 2:** Mental disorders distribution within the sample.

Mental disorders	Youth, *n*	Mother, *n*	Father, *n*
Depression	5	9	3
Bipolar disorder	1	1	0
Schizophrenia	0	1	1
Psychosis	1	1	0
Anorexia	1	0	0
Bulimia	2	0	0
Social phobia	0	1	0
Generalized anxiety disorder	1	4	1
Panic disorder	0	4	1
Agoraphobia	0	1	0
Somatoform disorder	1	0	0
Posttraumatic stress disorder	1	5	2
Obsessive-compulsive disorder	2	0	1
Attention deficit hyperactivity disorder	1	2	2
Conduct disorder	1	0	0
Alcohol addiction	0	2	2
Drug addiction	0	1	0
Borderline personality disorder	2	2	0
Autism	1	0	0
Unknown	0	2	2

**Table 3:** Primary outcome: YSR 11-18R sum scores.

Timepoint	IG mean (SD)	*n*	CG mean (SD)	*n*
At t1	77.8 (34.27)	10	79.42 (42.46)	12
At t2	57 (13.67)	6	79 (38.64)	7
At t3	84.33 (28.92)	6	81.56 (34.96)	9
At t4	73.71 (33.62)	7	72.5 (33.70)	8

**Table 4:** Secondary outcome: sum scores.

Outcome	Timepoint	IG mean (SD)	*n*	CG mean (SD)	*n*
WHO-5 (60)	At t1	10.8 (6.83)	10	10.08 (5.52)	12
	At t2	11.8 (4.44)	5	11.86 (6.91)	7
	At t3	8.2 (4.38)	5	12 (5.63)	9
	At t4	10 (5.16)	7	11 (6.93)	8
OSSQ-3	At t1	7.9 (2.13)	10	10.75 (1.86)	12
	At t2	9.2 (1.92)	5	11.57 (2.57)	7
	At t3	7.25 (11.33)	4	11.33 (2.18)	9
	At t4	7 (1.53)	7	11.25 (2.55)	8
Brief COPE Problem-focused coping (58)	At t1	17.1 (4.86)	10	17.58 (4.81)	12
	At t2	15 (1.22)	5	17.86 (5.18)	7
	At t3	13.25 (2.06)	4	18.13 (4.32)	8
	At t4	16.43 (2.15)	7	17.5 (3.78)	8
Brief COPE Emotion-focused coping (58)	At t1	25.3 (5.74)	10	26.08 (4.80)	12
	At t2	24 (5.43)	5	26.43 (5)	7
	At t3	23 (3.74)	4	26.88 (2.42)	8
	At t4	23.14 (2.79)	7	26.38 (2.2)	8
Brief COPE Avoidant coping (58)	At t1	13.2 (3.33)	10	12.42 (4.58)	12
	At t2	11.6 (2.19)	5	11.14 (3.44)	7
	At t3	14.25 (4.03)	4	11.75 (3.05)	8
	At t4	13.29 (3.15)	7	12.63 (2.39)	8
SES (59)	At t1	25.8 (6.12)	10	25.17 (6.62)	12
	At t2	23.8 (1.79)	5	26.49 (7.11)	7
	At t3	24.75 (6.4)	4	25.25 (5.44)	8
	At t4	21.57 (7.46)	7	24.13 (9.13)	8
WAI-I Bond	At t3	3.56 (1.01)	4		
	At t4	3.29 (1.07)	7		
WAI-I Task and goal	At t3	2.91 (0.51)	4		
	At t4	2.57 (0.57)	7		

### 3.2 Intervention usage and dropouts

From the *n* = 10 participants in the IG, *n* = 2 (20%) did not start the intervention, *n* = 4 (40%) finished one module, *n* = 1 (10%) finished five modules, and *n* = 3 (30%) finished all eight modules.

Dropouts were differentiated into study, assessment, and intervention dropouts. Assessment dropouts at t3 were *n* = 7 (31.81%), and complete study dropouts at t3 were *n* = 2 (9.09%). Of the *n* = 10 participants in the IG, *n* = 6 (60%) were classified as intervention dropouts, due to not having finished at least half of the available modules.

### 3.3 Negative effects

Any statistical analyses of the negative effects data could not be done due to the small sample size. A descriptive evaluation of the NEQ (34) showed that *n* = 4 participants attributed *n* = 19 negative effects to the intervention: *unpleasant memories resurfacing* (*n* = 1 at t1, *n* = 2 at t2, *n* = 4 at t3), *did not always understand the therapist* (*n* = 1 at t1), *feelings that the treatment did not produce any results* (*n* = 1 at t1, *n* = 2 at t2), *experiencing more unpleasant feelings* (*n* = 1 at t2, *n* = 1 at t3), *thoughts that it would be better if not exist anymore or should take my life* (*n* = 1 at t2), *stopped thinking that things could get better* (*n* = 1 at t2, *n* = 1 at t3), *did not always understand the treatment* (*n* = 1 at t2), *feeling ashamed in front of other because of treatment* (*n* = 1 at t3), and *my expectations for the therapist were not fulfilled* (*n* = 1 at t2). How negatively these incidents affected the participants was predominantly rated as 1 or 2 (from 1 “not at all” to 5 “extremely”), except for one incident rated as 4 (*did not always understand the therapist*). The one participant who mentioned *that it would be better if he or she doesn't exist anymore* was asked additional questions to evaluate the severity of the suicidal ideation, and it was determined that no intention to act was imminent.

In total, five different participants (IG = 4, CG = 2) reported suicidal ideation within any of the used assessment scales during all four measurement time points. However, all five participants reported that they only *occasionally or slightly* had these thoughts. These answers triggered automatic emails with information and help, but the answers were below the predetermined threshold that required further steps according to the defined process for severe adverse events.

## 4 Discussion

The present two-armed, multicenter, cluster-randomized controlled trial was designed to investigate the clinical and cost-effectiveness of the Internet- and mobile-based intervention iCHIMPS between an intervention group and a treatment-as-usual control group. However, due to unsuccessful recruitment (*n* = 22), which fell far short of the *a priori* defined target sample size of 306 participants, we were unable to meaningfully conduct any of the planned analyses. Unfortunately, recruitment difficulties, as observed in this trial, are common in this field of research (21), as are ambiguous results regarding the effectiveness of mental health IMIs for children and adolescents (2, 3, 5, 8, 19, 21). Along with this, underreporting of potential negative effects of the interventions has also been noted (8). Therefore, the remainder of the discussion focuses on the reasons why this trial failed to achieve its recruitment goals, how it relates to previous research, and what these insights may imply for IMI research with children and adolescents in general. In this way, the evaluation of this failed trial might offer valuable lessons for future research projects.

Drawing on insights from the existing literature and the authors' interpretation of how the trial was conducted and contextualized, several potential factors are proposed that may have contributed to the trial's inability to achieve its intended outcomes. These have been categorized into three main areas: study-related, population-related, and intervention-related challenges.

First, study-related problems pertained to all aspects of the study design. One major barrier to successful recruitment was the implementation of too many separate arms within the overall research project (32). In our case, four trials were ambitiously designed, which led to competition for limited recruiting resources at the same sites, which were responsible for recruiting from the same or similar populations. Consecutive recruiting was planned but could not be realized due to a general lack of eligible participants, partly attributable to the COVID-19 pandemic and its consequences. While the in-person trials eventually reached their (reduced) target sample size, the iCHIMPS trial had to be terminated prematurely. Another factor that may have hindered recruitment appears to be the fragmented structure of the healthcare system in this context, with separate adult and child and adolescent mental health facilities. For this trial, a research strategy was implemented that relied primarily on a fixed number of adult mental health clinics, as the family clusters first needed to include a mentally ill parent. However, study staff were employed at either adult or child/adolescent clinics, and collaboration between departments was inconsistent. The COVID-19 pandemic further limited recruitment by closing the already scarce cross-department pathways in the recruitment strategy of this trial. We therefore suggest that future trials carefully evaluate the feasibility of proposed recruitment pathways in advance and adjust the complexity of the research design accordingly. Unfortunately, even broadening the recruitment strategy to include a wider and more diverse set of recruiting pathways, and sites did not change the recruitment outcome, leading to the next cluster of problems.

Second, population-related problems concerned all aspects of the chosen target population. The iCHIMPS IMI targeted families with at least one mentally ill parent and one child. In the literature, this population is considered an at-risk group (14, 16); however, help-seeking behavior within this group is often observed to be low (35). Research indicates that these families may avoid available services due to feelings of failure, shame, guilt, or fear of losing custody of their children (35). More generally, recruiting minors for research is especially challenging, due to the fact that parents or legal guardians need to provide informed consent on behalf of their children. These emotional and procedural barriers may reduce recruitment outcomes and bias the sample (21, 36, 37). Taken together, families with mentally ill parents are a hard-to-reach population, which may have contributed to the recruitment difficulties observed in this trial. Nonetheless, this information was available prior to the start of the study and may not have been fully integrated into the design or the interventions, as will be elaborated on in the next section.

Third, intervention-related problems concerned all aspects of the implemented intervention. The iCHIMPS IMI was designed to be used solely by the children, rather than the entire family. While previous research suggests that family-centered interventions may be the most suitable for this population (38–40) or even more effective than individually focused interventions (39, 40), the present trial specifically aimed to evaluate an adolescent-focused digital intervention. Informal feedback from some adolescents during recruitment suggested that they did not perceive themselves as the primary target of the intervention, and questioned their participation. This may indicate that child-focused interventions could be more effective when embedded within broader family-based models rather than standalone treatments. Future research should explore the potential of combining family-centered interventions with adjunct programs for the children, and in this case, perhaps IMIs could still be a feasible solution. Another issue with the iCHIMPS IMI was the age appropriateness of the intervention, which may have impacted both the uptake and the adherence. Despite incorporating video and audio elements, the intervention remained text-heavy, as noted during the think-aloud development process. Previous research with adult samples has indicated that IMI studies tend to recruit an overrepresentation of well-educated, middle-aged female participants (41, 42). This self-selection (41) may drive a preference for theoretical content, reinforcing feedback loops in intervention design that favor complexity. In the context of adolescents, the text-heavy design of iCHIMPS could have negatively impacted adherence, though the small sample size prevents meaningful conclusions. A broader issue lies at the intersection of population and intervention: This raises the important and still open question of whether IMIs are a suitable treatment format for adolescents with mentally ill parents. On the surface, it seems logical that digital interventions would appeal to digitally savvy youth. However, evidence indicates that IMIs are generally less effective for children and adolescents compared with adults (3–6, 8). It is also possible that, contrary to expectations, digitally savvy adolescents may not prefer interventions resembling digital entertainment, especially when the look appears to be of lower quality. The overlap between intervention and entertainment may even hinder serious engagement. Moreover, developmental factors, such as whether adolescents have sufficient intrinsic motivation and self-management skills for this type of intervention, must be considered. All of these questions will need to be answered in future research, guiding us from the present trial to the broader topic of IMI research with children and adolescents in general.

The last point warrants further elaboration due to its significance beyond the present trial. Current literature (3, 43) sometimes differentiates between the effectiveness of IMIs for children (usually defined as up to 10 years) and adolescents (10–18 years) with unclear and mixed findings and a notable lack of studies focused solely on children (7, 43, 44). When comparing children and adolescents combined to adults, the literature shows fewer studies, smaller effect sizes, and much more ambiguous findings for minors (2–5, 8), while adults consistently benefit from IMIs (3, 5). This raises the question of whether IMIs simply are not effective, less effective, or not effective enough for children and adolescents. Alternatively, it is possible that research and intervention development have once again failed to design interventions truly tailored to children and adolescents, instead merely translating what has been shown to work for adults. These considerations point to two potential directions for future research and ethical reflection. One is to abandon IMI research for younger populations due to insufficient effectiveness, in line with the principle of beneficence (45, 46). The other is to recognize the potential of IMIs, their scalability, cost-effectiveness, and time and location independence (41), while addressing the challenges of adapting the current “adult framework” to younger users. Doing so may improve engagement, adherence, and ultimately, effectiveness. Importantly, differences in effect sizes between age groups are also observed for traditional face-to-face psychotherapy (3, 47–50). As highlighted in prior research, this issue may not be specific to IMIs but could reflect broader challenges associated with effectively treating younger populations.

Endorsing the second path, adapting current IMIs for children and adolescents to help close the treatment gap (51, 52), raises the question of how this can be achieved. How can IMIs be designed to increase attractiveness, engagement, and adherence? Previous research has identified two main themes to address these challenges: person-specific and intervention-specific factors (53). Person-specific factors include connectedness to the intervention (e.g., interaction with others to reduce isolation), trust in the privacy and anonymity, credibility and validity, and motivation driven by perceived usefulness and interest, while avoiding content that feels too generic (53). Intervention-specific factors involve the acceptability of features and language, reducing text, incorporating more media content, customizability, peer connection, ease of use, intuitiveness, age appropriateness, and accessibility (53). These suggestions highlight a distinct set of needs and preferences in children and adolescents, many of which should be integrated into future designs. One research field aiming to enhance IMIs through technological features is the field of persuasive design (29). However, most research in this area focuses on adults, with limited emphasis on children and adolescents. In the future, basic research into the dissemination and delivery of digital treatment should differentiate more clearly between adults and younger populations. Step by step, we should move beyond a “one-size-fits-all” approach, which assumes children are just small adults, and toward more tailored solutions that reflect the developmental, motivational, and contextual realities of children and adolescents.

Another important question is whether interventions with unclear effectiveness for children and adolescents might cause harm. This concern aligns with the principle of non-maleficence (45, 46). Current research indicates that negative effects of mental health IMIs for children and adolescents are often underreported but present (8). Evidence suggests that negative effects do not appear significantly more frequent in IMI groups compared with active control groups; however, some negative effects are clearly attributable to intervention use when compared with passive control groups (8). In the current trial, negative effects were also assessed, and five participants attributed 19 negative effects to the intervention use. Nearly half of these were related to the resurfacing of negative memories and emotions, effects that may be inherent to many mental health interventions and might be expected. Some research even suggests such experiences may mediate positive therapy outcomes (54). However, other research shows no clear link between negative effects and symptom reduction (55, 56), while some indicate that negative effects may predict poorer treatment results (57). Further studies should distinguish between negative effects that drive therapeutic change and those that impede it. Importantly, nearly half of the remaining negative effects reported in the present study were linked to a lack of trust in the therapist or the treatment and a lack of understanding of the treatment. While the small sample size limits generalizability, these specific effects appear directly related to the design of the intervention and underscore the need to adapt IMIs to the developmental needs and preferences of children and adolescents, as discussed in the previous section.

A strength of the study was its large-scale, multisite design with multiple intervention arms, which was intended to enhance generalizability and support broad comparative analyses. However, this potential could not be fully realized due to significant recruitment challenges. Recruitment capacity was overestimated at several sites, resulting in insufficient sample sizes and limited statistical power. The inclusion of multiple arms may have further reduced the ability to evaluate each intervention with sufficient validity. Additionally, the interventions may not have been adequately tailored to the needs of the target population, potentially impacting engagement. Recruitment efforts were also not systematically tracked, limiting insight into the effectiveness of individual recruitment strategies. Future studies would benefit from a more focused design, better-adapted interventions, and pretested, monitored recruitment pathways.

## 5 Conclusion

The present trial failed to achieve its recruitment goals, and consequently, no planned analyses could be conducted. Nonetheless, valuable lessons can be drawn from it. Future IMI research targeting children of mentally ill parents should thoroughly assess recruitment strategies and align the complexity of both research design and interventions with the needs and preferences of the target population. These lessons are also relevant for IMI research involving children and adolescents in general. IMIs should not follow a one-size-fits-all approach, as children and adolescents are not simply small adults without distinct needs and preferences. Furthermore, evaluating negative effects alongside effectiveness provides a more comprehensive understanding of the intervention impact, following the principles of beneficence and non-maleficence.

## Data Availability

The raw data supporting the conclusions of this article will be made available by the authors, without undue reservation.
